# 15- Deoxy-Δ12,14-prostaglandin J2 upregulates COX-2 expression and PGE_2_ in mouse mesangial cells through a mechanism independent of PPARγ

**DOI:** 10.3389/fphar.2026.1924049

**Published:** 2026-09-11

**Authors:** Bo Yu, Yuping Zhang, Zhenqi Hu, Xuyan Fu, Xinyi Shen, Xinghao Rong, Guangrui Yang

**Affiliations:** 1 School of Clinical Medicine, Shanghai University of Medicine and Health Sciences, Shanghai, China; 2 School of Basic Medicine, Shanghai University of Medicine and Health Sciences, Shanghai, China

**Keywords:** 15-deoxy-δ12, 14-prostaglandin J2, cyclooxygenase-2, glomerular, mesangial cells, PPARγ, prostaglandin E2

## Abstract

15-Deoxy-Δ12,14-prostaglandin J_2_ (15d-PGJ_2_), an endogenous cyclooxygenase-derived lipid mediator and natural ligand of peroxisome proliferator-activated receptor gamma (PPARγ), plays important roles in inflammatory regulation and renal pathophysiology. However, the effect of 15d-PGJ_2_ on cyclooxygenase-2 (COX-2) expression in mesangial cells and the involvement of PPARγ in this process remain unclear. In this study, we investigated the regulation of COX-2 expression and prostaglandin E_2_ (PGE_2_) production by 15d-PGJ_2_ in primary cultured mouse mesangial cells and determined the role of PPARγ using pharmacological and genetic approaches. Treatment with 15d-PGJ_2_ significantly increased COX-2 mRNA and protein expression, accompanied by a marked elevation of PGE_2_ production. Pharmacological inhibition of PPARγ using the selective antagonist GW9662 did not affect 15d-PGJ_2_-induced COX-2 expression or PGE2 production. Similarly, overexpression of dominant-negative PPARγ, siRNA-mediated knockdown, and Cre-mediated genetic deletion of PPARγ failed to attenuate the induction of COX-2 by 15d-PGJ_2_. Functional reporter assays confirmed effective modulation of PPARγ transcriptional activity under these conditions, indicating that the lack of effect was not due to insufficient inhibition. These findings demonstrate that 15d-PGJ_2_ induces COX-2 expression and enhances PGE_2_ production in mouse mesangial cells via a mechanism independent of PPARγ. This PPARγ-independent action suggests alternative signaling pathways through which endogenous lipid mediators regulate inflammatory responses in the kidney. Our results provide new insight into the complex role of prostaglandin metabolites in renal inflammatory signaling and highlight the importance of receptor-independent actions of electrophilic lipid mediators.

## 1 Introduction

Peroxisome proliferator-activated receptor γ (PPARγ) is a ligand-activated nuclear transcription factor belonging to the nuclear hormone receptor superfamily. PPARγ binds, as a heterodimer with retinoid X receptor (RXR) (Fang et al., 2021), to peroxisome-proliferator response element (PPRE) located in the promoters of numerous target genes that are involved in various physiological or pathophysiological processes including metabolism, inflammation, oxidative stress, tumor and development (Thakur et al., 2023; Chen et al., 2023; Hernandez-Quiles et al., 2021; Guan, 2004; Colca et al., 2014; Zhou et al., 2020). It is well established that PPARγ is constitutively expressed in glomerular mesangial cells and plays protective roles in the progression of nephropathy (Agrawal et al., 2021; Kökény et al., 2020). Agonists of PPARγ include synthetic compounds such as antidiabetic thiazolidinediones, and natural ligands such as prostaglandin D_2_ (PGD_2_) metabolite 15-deoxy-Delta12,14-prostaglandin J_2_ (15d-PGJ_2_) (Guan and Breyer, 2001). The endogenous ligand, 15d-PGJ_2_, was shown initially to have a major function in adipocyte differentiation and glucose homeostasis (Chawla et al., 1994; Tontonoz et al., 1994), and has been reported to reverse the phenotypic changes of mesangial cells and regulate cell proliferation and death (Rovin et al., 2002; Asano et al., 2000; Stamatakis et al., 2006).

Cyclooxygenase (COX) is a key enzyme in prostaglandin (PG) synthesis, catalyzing the conversion of arachidonic acid to PGH_2_, a common precursor for bioactive prostaglandins including PGJ_2_ (Wang et al., 2018). Among its two major isoforms, COX-1 is constitutively expressed to maintain physiological PG functions, while COX-2 is an immediate-early response gene rapidly induced during inflammatory disorders and oxidative stress (Grosser et al., 2010). Previous studies indicate that PPARγ ligands can suppress COX-2 expression in various cell types, such as epithelial cells (Scoditti et al., 2010), synoviocytes (Tsubouchi et al., 2001), monocytes (Pontsler et al., 2002), and macrophage-like differentiated cells (Inoue et al., 2000). Conversely, induction of COX-2 by these ligands has also been reported in epithelial cells and synovial fibroblasts (Kalajdzic et al., 2002). Studies utilizing the irreversible PPARγ-selective antagonist GW9662 and dominant-negative constructs suggest that this regulation is highly cell-type-specific and involves both PPARγ dependent and independent mechanisms (Scoditti et al., 2010; Konger et al., 2010; Ayoub et al., 2009).

In this context, cyclopentenone prostaglandins like 15d-PGJ_2_ are increasingly recognized not merely as classical PPARγ ligands, but as specialized bioactive lipid mediators that actively modulate inflammation and promote tissue homeostasis (Macia Guardado et al., 2025; Park, 2026; Peh and Chen, 2025). Recent evidence highlights that such lipid mediators can regulate inflammatory gene expression through diverse signaling pathways beyond classical nuclear receptor activation (Li et al., 2023; Hinz et al., 2003; Bianchi et al., 2005; Lee et al., 2007). In glomerular pathophysiology—such as in diabetic nephropathy, lupus nephritis, and IgA nephropathy—the COX-2-derived PGE_2_ pathway plays a pivotal role in driving mesangial expansion and inflammatory progression. Although synthetic PPARγ agonists (e.g., pioglitazone) and 15d-PGJ_2_ have been explored in animal models of nephropathy, the double-edged sword nature of 15d-PGJ_2_ remains a challenge. Crucially, 15d-PGJ_2_ exhibits a unique concentration-dependent bimodal activity: acting as a potent PPARγ agonist at low (nanomolar) concentrations, while imposing receptor-independent electrophilic stress at higher (micromolar) concentrations. However, the precise role of PPARγ in mediating 15d-PGJ_2_-induced COX-2 expression in mesangial cells remains incompletely understood.

Therefore, we hypothesized that at micromolar concentrations, 15d-PGJ_2_ upregulates COX-2 and PGE_2_ in mouse mesangial cells through its electrophilic α, β-unsaturated carbonyl moiety rather than through canonical PPARγ activation, and that this effect would consequently be resistant to PPARγ antagonism, dominant-negative interference, siRNA knockdown, and genetic deletion. Using complementary pharmacological inhibition, dominant-negative interference, siRNA-mediated knockdown, and genetic deletion approaches, we demonstrate that 15d-PGJ_2_ induces COX-2 expression and PGE_2_ production through a PPARγ-independent mechanism. These findings provide new insight into receptor-independent signaling pathways of endogenous prostaglandin metabolites in renal cells.

## 2 Materials and methods

### 2.1 Chemicals and reagents

15d-PGJ_2_, GW9662 and Rabbit anti-human COX-2 antibody were obtained from Cayman Chemical Company (Ann Arbor, MI). Mouse anti-human PPARγ and α-tubulin antibodies were from Santa Cruz Biotechnology (Santa Cruz, CA) and Sigma (St. Louis, MO), respectively. All the corresponding secondary antibodies and other reagents for Western blotting were from Zhongshan Golden Bridge Company (Beijing, China). Real-time PCR was performed in PTC-200 (MJ Research, Waltham, MA) with reagents obtained from Tiangen (Beijing, China). The wild-type and dominant negative PPARγ plasmid, PPRE luciferase plasmids and the Cre adenovirus were kindly provided by Dr. Youfei Guan (Dalian Medical University, China). The PPARγ siRNA was constructed by Kangcheng Company (Shanghai, China).

### 2.2 Primary culture of mouse mesangial cells

3-week-old PPARγflox/flox (PPARγf/f) mice and C57BL/6 mice were used for primary mesangial cell culture as previously described with modifications (Wu et al., 2004). Briefly, renal cortex was minced and incubated in a 50 mL-tube containing 5 mL of 1 mg/mL Collagenase II in PBS at 37 °C. After 1-h gentle shaking, the digested cortexes were poured in a 40 µm sieve and smashed, followed by two washes using 20 mL of PBS. Then invert the sieve and set it on a culture dish and wash the sieve again. The flow through containing glomeruli was collected and centrifuged at 1000 rpm for 10 min. Discard The supernatant was discarded, and the cell pellet was harvested in DMEM containing 20% FBS, streptomycin (100 mg/mL) and penicillin (100units/mL). Cells were grown at 37 °C in a humidified atmosphere of 95% air/5% CO^2^. The cells were left undisturbed for 5 days, and the culture medium was exchanged every 2 days thereafter.

Before experiments, cells were seeded into 6-well plates at 1 × 10^5^/well. After confluence, the medium was exchanged with serum-free medium for 24 h to maintain the cells in a quiescent, non-catabolic state for extended time periods in the subsequent experiments. Then, the cells were treated with 10 μM GW9662 for 1 h prior to the addition of 10 μM 15d-PGJ_2_ for indicated time. To construct a PPARγ knockout cell line, the mesangial cells from PPARγ^f/f^ mice were infected with adenovirus expressing Cre recombinase (Cre-Ad) at a multiplicity of infection (MOI) of 100 as determined with an adenovirus expressing GFP (GFP-Ad). Two days after infection, fresh growth media was changed for the successive 15d-PGJ_2_ treatment.

### 2.3 Reverse transcription PCR (RT-PCR)

Total RNA was extracted from cultured mesangial cell using RNAtrip Reagent (Applygen, China). 2 μg total RNA were reverse transcribed to cDNA using hexadeoxynucleotide random primers and Moloney murine leukemia virus reverse transcriptase (Promega, Madison, WI) according to manufacturer’s instructions. The cDNAs were used as templates for RT-PCR and quantitative RT-PCR. The primer sets for COX-2 (cyclooxygenase-2) are 5′-AGA​AGG​AAA​TGG​CTG​CAG​AA-3’ (sense) and 5′- GCT​CGG​CTT​CCA​GTA​TTG​AG-3’ (antisense), for PPARγ (peroxisome proliferator-activated receptor gamma) 5′-GTTTGCTGTGAA GTTCAATGC-3’ (sense) and 5′-GTC​TGT​CTC​CGT​CTT​CTT​GAT-3’ (antisense), and for α-tubulin (alpha-tubulin) 5′-AGC​CAT​GTA​CGT​AGC​CAT​CC-3’ (sense) and 5′-GCTGTGGTG GTGAAGCTGTA-3’ (antisense). The PCR reactions were carried out at 94 °C for 5 min, followed by 35 cycles of 94 °C for 30 s, 60 °C for 30 s, and 72 °C for 30 s with a final extension at 72 °C for 5 min.

### 2.4 Western blot analysis

Cellular proteins were extracted as previously (Yang et al., 2006). Proteins were separated by SDS-PAGE and transferred to nitrocellulose. Immunoblotting was performed using antibodies directed against PPARγ, COX-2, and α-tubulin in 1 × PBS containing 0.05% (v/v) Tween-20% and 5% (w/v) BSA. After incubation in horseradish peroxidase-conjugated secondary antibodies, the blots were washed and applied to enhanced chemiluminescent detection reagent. The signals were revealed using Kodak film and quantified by densitometry.

### 2.5 Prostaglandin E_2_ enzyme immunoassay

Cells were plated in six-well plates, following treatments, the harvested supernatants were centrifuged to remove particulate matters and assayed for PGE content by a PGE-specific EIA kit following the manufacturer’s instruction (Cayman Chemical, Ann Arbor, MI).

### 2.6 PPRE reporter assay

Transient transfection was performed in cultured mesangial cells using High Efficient Transfection Reagent (Invitrogen, Carlsbad, CA). Cells were transfected with a wild-type PPARγ construct or a dominant-negative PPARγ construct, together with a PPRE-luciferase reporter plasmid and a β-galactosidase vector. An empty vector pcDNA3.1 was used as an internal control. PPRE luciferase was measured 24 h after transfection. β-galactosidase activity was measured for normalization.

### 2.7 siRNA transfection

We selected siRNA corresponding to a conserved sequence (nucleotides 545–563; Accession No. NM_011146) to target transcript of the mouse PPARγ gene. We also used a scramble siRNA whose sequence did not match any mouse genomic sequence. Transfection of siRNA was performed on mouse mesangial cells at 60%–70% confluence. 2 μL of High Efficient Transfection Reagent (Invitrogen, Carlsbad, CA) was mixed with 100 uL of Opti-MEM medium (Invitrogen Carlsbad, CA) and incubated for 5 min at room temperature. 5 μg siRNA plasmid were diluted in another 100 uL of Opti-MEM medium, mixed with the diluted transfection reagents and incubated for another 15 min, then the mixture was added to the cells (2 mL of DMEM medium with 5% FBS) and lasted for transfection. Scramble siRNA controls were used in parallel. After 48 h incubation, the activity of GFP was measured to evaluate transfection efficiency, and then cells were treated with or without 15d-PGJ_2_ for another 4 h before lysis.

### 2.8 Statistical analysis

All data are presented as mean ± SEM. Statistical analyses were performed using Student’s t-test or one-way analysis of variance (ANOVA) where appropriate. Differences were considered statistically significant at P < 0.05.

## 3 Results

### 3.1 15d-PGJ_2_ induces COX-2 expression and PGE_2_ production in mouse mesangial cells

**FIGURE 1:**
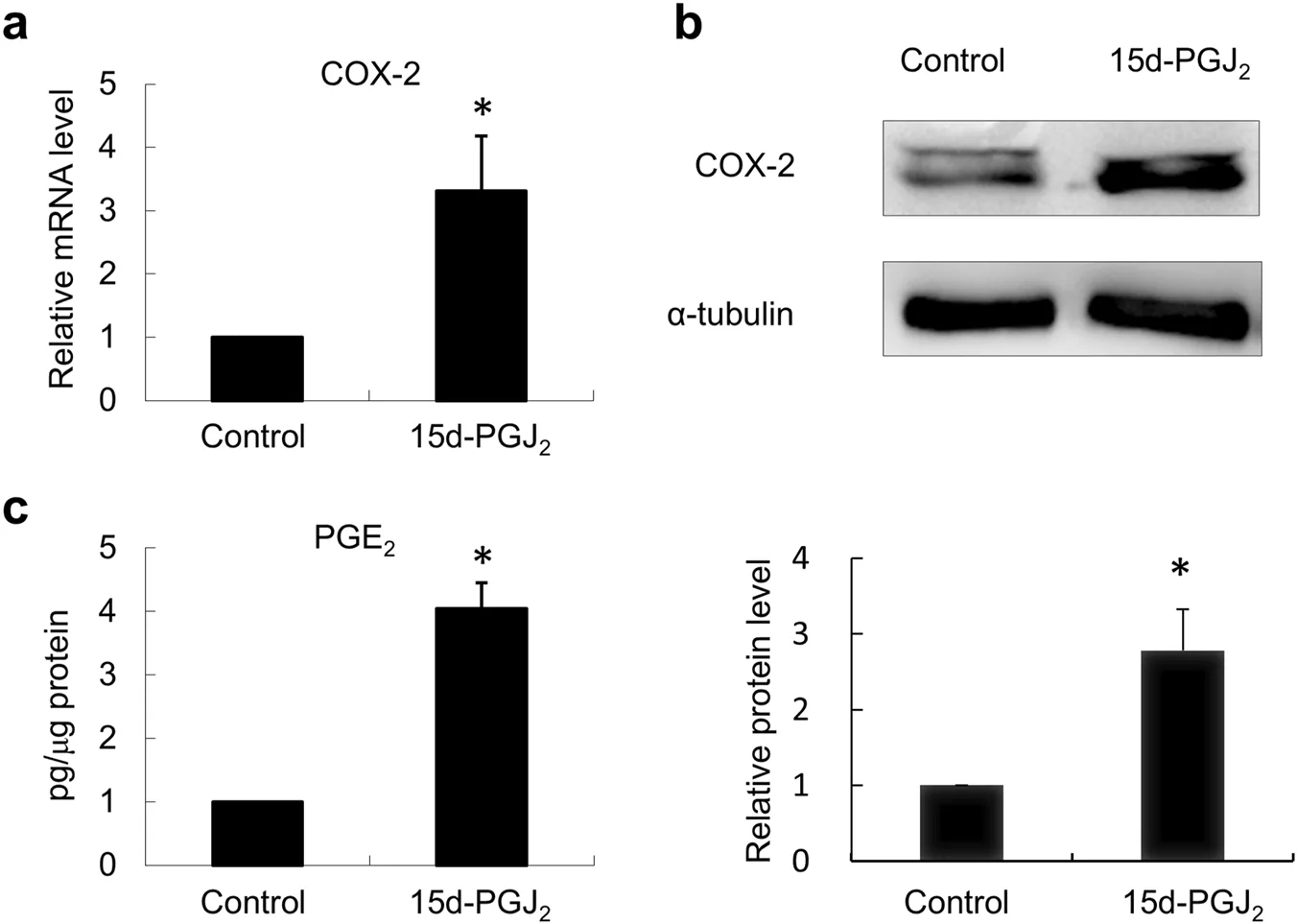
15d-PGJ2 upregulates mRNA **(a)** and protein **(b)** expression of COX-2 and increases PGE2 production **(c)** in mouse mesangial cells (*p < 0.05 versus control, n = 3).

We treated primary cultured mouse mesangial cells with 10 uM 15d-PGJ_2_ for 4 h and found that both mRNA and protein level of COX-2 were increased (Figures 1a,b). To determine whether the 15d-PGJ_2_ induced COX-2 is functional, we measured the levels of PGE_2_, one of major products of COX-2, in the culture medium. As the data shown, 15d-PGJ_2_ treatment resulted in a four-fold increase in PGE_2_ production (Figure 1c).

### 3.2 Induction of COX-2 by 15d-PGJ_2_ cannot be blocked by PPARγ antagonist GW9662

**FIGURE 2:**
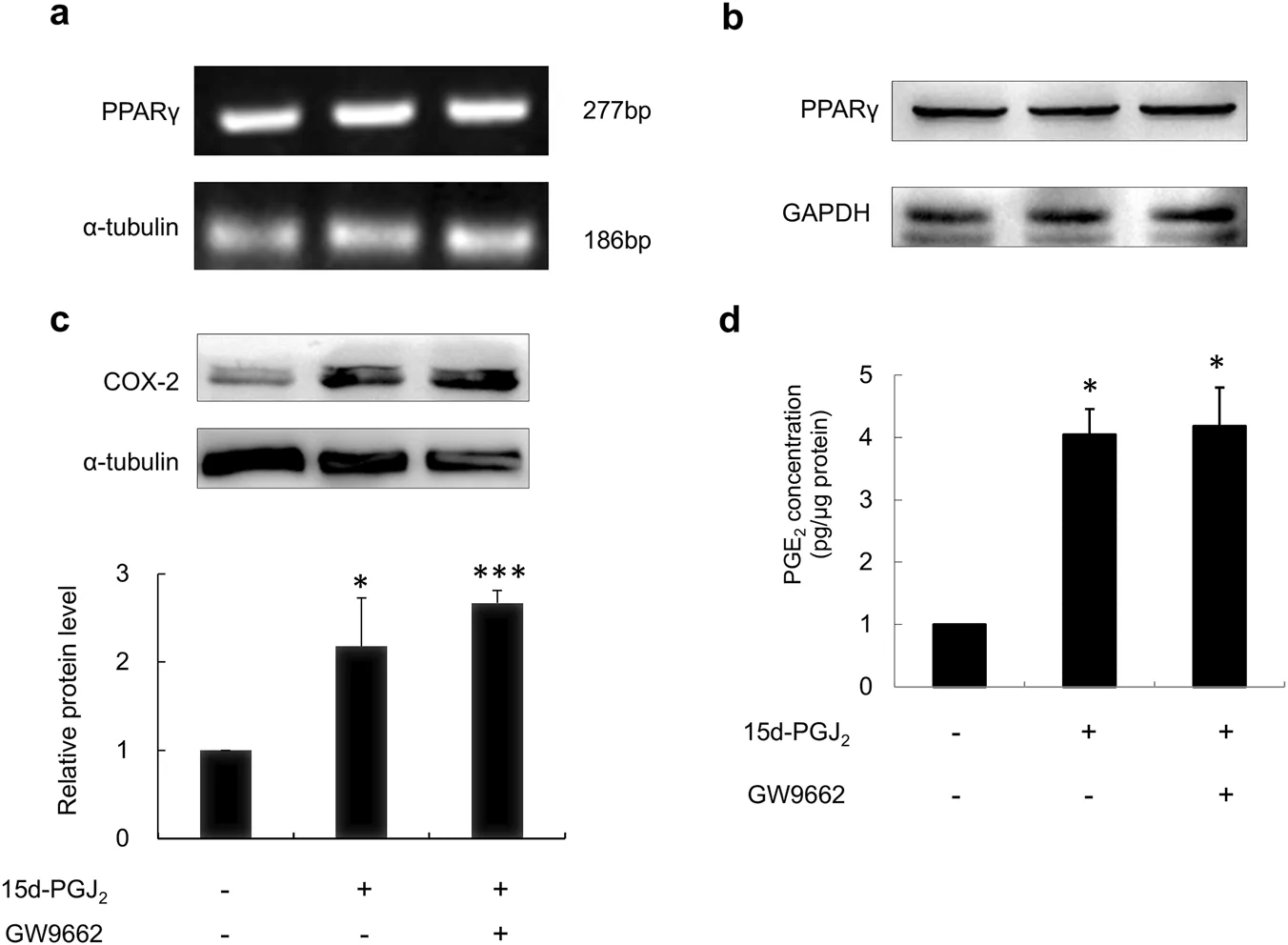
PPARγ antagonist GW9662 has no effect on the induction of 15d-PGJ2 on COX-2 expression and PGE2 production. **(a)** PPARγ mRNA constitutively expressed in cultured mouse mesangial cells. **(b)** PPARγ protein constitutively expressed in cultured mouse mesangial cells. **(c)** PPARγ antagonist GW9662 has no effect on 15d-PGJ2-induced COX-2 protein expression (*p < 0.05; ***p < 0.001 versus control, n = 3). **(d)** PPARγ antagonist GW9662 has no effect on 15d-PGJ2-induced PGE2 production (*p < 0.05 versus control, n = 3).

15d-PGJ_2_ has been identified as a natural ligand for PPARγ. In order to determine whether the induction of COX-2 by 15d-PGJ_2_ is PPARγ-dependent, we first examined the expression of PPARγ in the mesangial cells. As shown in Figure 2a, PPARγ mRNA was detected by RT-PCR, yielding an expected 277 bp fragment in three preparations of mesangial cells. Western blot analysis showed that PPARγ protein was also constitutively expressed in mesangial cells (Figure 2b). Next, we investigated whether the induction of COX-2 by 15d-PGJ_2_ can be blocked by PPARγ antagonist GW9662. As shown in Figures 2c,d, GW9662 was not able to inhibit 15d-PGJ_2_-induced COX-2 protein expression or PGE_2_ production.

### 3.3 PPARγ dominant negative cannot reverse the induction of COX-2 by 15d-PGJ_2_

**FIGURE 3:**
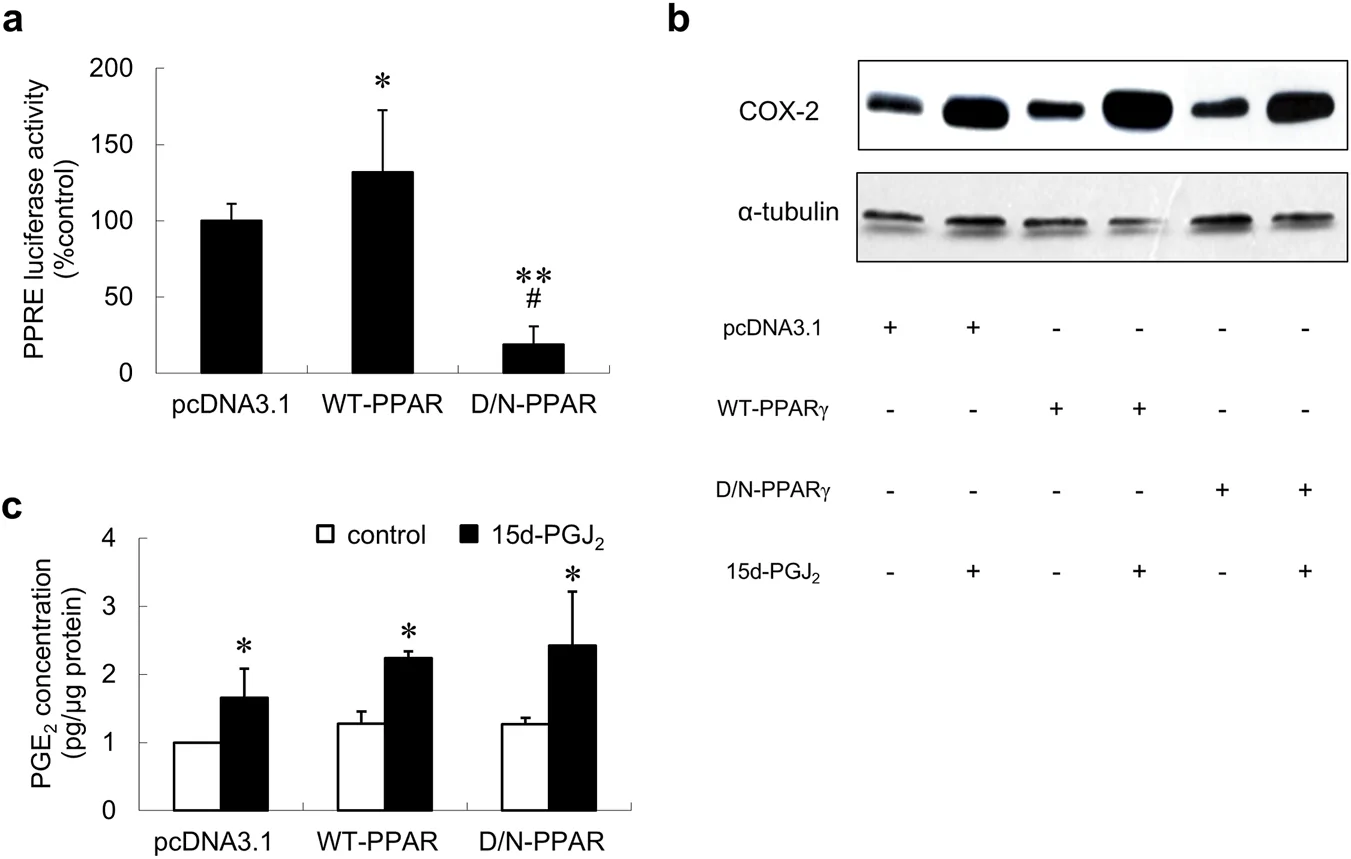
PPARγ dominant negative has no effect on the induction of 15d-PGJ2 on COX-2 expression and PGE2 production. **(a)** WT-PPARγ increases while D/N-PPARγ decreases PPRE luciferase activity; (*p < 0.05; **p < 0.01 versus control; #p < 0.05, versus WT-PPARγ, n = 3). **(b)** Neither WT-PPARγ nor D/N-PPARγ has effect on 15d-PGJ2-induced COX-2 protein expression. **(c)** Neither WT-PPARγ nor D/N-PPARγ has effect on 15d-PGJ2-induced PGE2 production (*p < 0.05 versus control, n = 3).

To provide further evidence, we transfected the cells with plasmids expressing wild-type PPARγ (WT-PPARγ) or dominant negative PPARγ (D/N-PPARγ). This D/N-PPARγ harbors two mutations in trans-activation domain and is a potent inhibitor of PPARγ function. As shown in Figure 3a, the WT-PPARγ significantly increased the PPRE luciferase activity, while the D/N-PPARγ plasmid decreased the PPRE activity. Furthermore, as expected, neither wild-type nor dominant negative plasmid has effect on 15d-PGJ_2_-induced COX-2 expression (Figure 3b) and PGE_2_ production (Figure 3c).

### 3.4 PPARγ siRNA failed to reverse the induction of COX-2 by 15d-PGJ_2_

**FIGURE 4:**
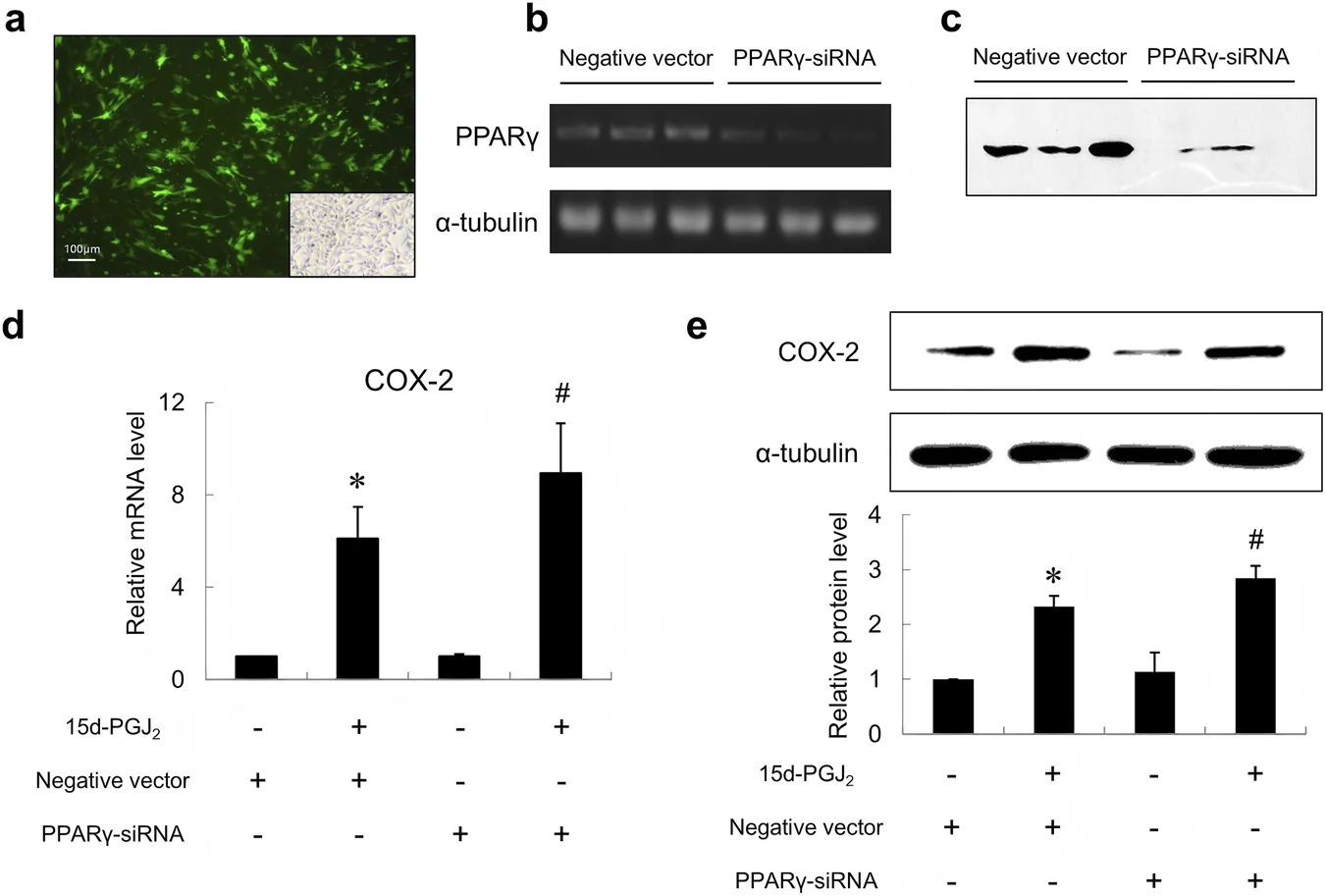
PPARγ siRNA has no effect on the induction of 15d-PGJ2 on COX-2 expression. **(a)** Transfection efficiency evaluated by GFP-positive cells (green fluorescence; inset: bright-field image; scale bar = 100 μm). Quantitative analysis of GFP-positive cells from multiple randomly selected fields indicated approximately 90% transfection efficiency. **(b)** PPARγ mRNA expression was decreased by PPARγ siRNA transfection. **(c)** PPARγ protein expression was inhibited by PPARγ siRNA transfection. **(d)** PPARγ siRNA has no effect on 15d-PGJ2-induced COX-2 mRNA expression. (*p < 0.05 versus negative vector only; #p < 0.05, versus PPARγ-siRNA only, n = 3) **(e)** PPARγ siRNA has no effect on 15d-PGJ2-induced COX-2 protein expression (*p < 0.05 versus negative vector only; #p < 0.05, versus PPARγ-siRNA only, n = 3).

We co-transfected a PPARγ siRNA plasmid and a GFP expression plasmid to the mesangial cells. Transfection efficiency was validated by the ratio of GFP positive cells. About 90% cells were transfected as shown in Figure 4a. Meanwhile both RNA (Figure 4b) and protein levels (Figure 4c) of PPARγ were significantly reduced by siRNA transfection. While this reduction failed to affect the induction of 15d-PGJ_2_ on COX-2 expression in either mRNA (Figure 4d) or protein (Figure 4e) level.

### 3.5 PPARγ gene knockout failed to abolish the induction of 15-dPGJ_2_ on COX-2 expression and PGE_2_ production

**FIGURE 5:**
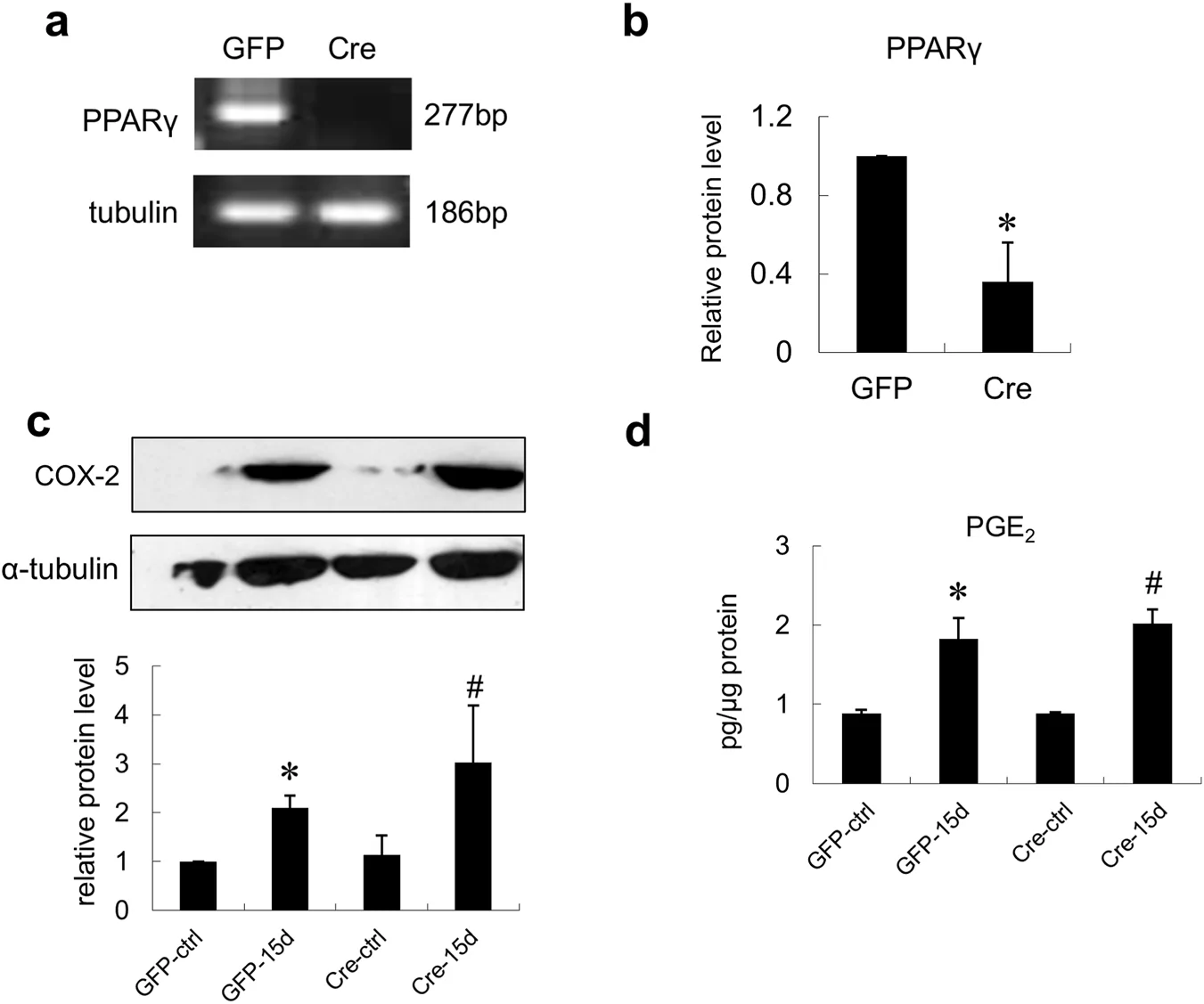
PPARγ gene knockout has no effect on the induction of 15d-PGJ2 on COX-2 expression and PGE2 production. **(a)** PPARγ mRNA expression was blocked by Cre adenovirus infection. **(b)** PPARγ protein expression was inhibited by Cre adenovirus infection. **(c)** Cre-mediated PPARγ deletion has no effect on 15d-PGJ2-induced COX-2 protein expression (*p < 0.05 versus GFP-ctrl; #p < 0.05, versus Cre-ctrl, n = 3). **(d)** Cre-mediated PPARγ deletion has no effect 15d-PGJ2-induced PGE2 production (*p < 0.05 versus GFP-ctrl; #p < 0.05, versus Cre-ctrl, n = 3).

Finally, we knocked out the PPARγ gene in the mesangial cells. Mesangial cells cultured from PPARγf/f mice were infected with Cre-expressing adenovirus (Cre-Ad). The adenovirus expressing GFP protein (GFP-Ad) was used as control. Cre-Ad infection dramatically decreased PPARγ expression in both mRNA and protein levels (Figures 5a,b). While 15d-PGJ_2_ induced COX-2 expression (Figure 5c) and PGE_2_ production (Figure 5d) remained comparable to that in GFP-Ad infected cells and Cre-Ad infected cells.

## 4 Discussion

Accumulating evidence suggests that cyclooxygenase (COX)-2 derived prostaglandin E_2_ (PGE_2_) participates in many renal pathological processes such as glomerulosclerosis (Komers and Epstein, 2002; Zhuang et al., 2018; Nguyen, 2006). The transcription factor peroxisome proliferator-activated receptor gamma (PPARγ) has been reported to play a protective role in progression of nephropathy (Wu et al., 2009; Zhang et al., 2020). 15d-PGJ_2_, an endogenous PPARγ ligand, also a COX metabolite, has been considered as a potent negative regulator of inflammatory responses and oxidative stress and a promising therapeutic agent of many inflammatory diseases (Bie et al., 2018; Liu et al., 2012). However, the pharmacological effect of 15d-PGJ_2_ on the expression of COX-2 and the consequent PGE_2_ production remains under debate. It is increasingly recognized that many PPARγ ligands can exert biological effects through both receptor-dependent and receptor-independent mechanisms, and that “PPARγ ligand” is not synonymous with “PPARγ-dependent effect” (Chang et al., 2025).

It was reported that 15d-PGJ_2_ inhibits cytokine-elicited PGE_2_ synthesis and COX-2 expression in many cell types, such as macrophages (Inoue et al., 2000), synovial fibroblasts (Tsubouchi et al., 2001), and rat mesangial cells (Sawano et al., 2002; Sánchez-Gómez et al., 2004). However, others showed that in contrast 15d-PGJ_2_ augments COX-2 expression and PGE_2_ synthesis in human osteoarthritic chondrocytes (Fahmi et al., 2002). Furthermore, in human mesangial cells, 15d-PGJ_2_ was reported to upregulate COX-2, while this upregulation of COX-2 did not result in increased PGE_2_ production (Reyes-Martin et al., 2008). While different cellular sources or species disparities may contribute to these discrepancies, a critical distinction likely lies in the preexisting cellular state. Previous studies often evaluated 15d-PGJ_2_ in the context of active inflammation (e.g., cytokine pre-stimulation), whereas our study investigated its direct effects on quiescent, unstimulated cells. In the present study, using cultured mouse glomerular mesangial cells, we found that 15d-PGJ_2_ can dramatically upregulate COX-2 expression at both mRNA and protein levels in mouse mesangial cells. Also, the PGE_2_ production was increased after 15d-PGJ_2_ treatment. Accumulating evidence from studies of patients, animal models, or cell models has shown that COX-2-derived PGE_2_ in the kidney is involved in the pathophysiological processes of various renal diseases, such as glomerulonephritis, interstitial nephritis, and renal cell carcinoma (Kömhoff et al., 2000; Reichman et al., 2001; Hirasawa et al., 2006; Chen et al., 2004; Yang et al., 2015). Our findings that 15d-PGJ_2_ increases COX-2 expression and PGE_2_ production in mouse mesangial cells emphasize the necessity of re-evaluation of the potency of 15d-PGJ_2_ in the treatment of renal glomerular diseases.

Regarding the underlying signaling mechanisms, our data provide compelling evidence that this induction is PPARγ-independent. Specifically (Fang et al., 2021): The selective PPARγ antagonist GW9662 (Thakur et al., 2023); PPARγ dominant negative construct (Chen et al., 2023); PPARγ siRNA; and (Hernandez-Quiles et al., 2021) PPARγ loss-of-function had no effect on 15d-PGJ_2_ induced COX-2 expression and PGE_2_ production. There is evidence that several intracellular signaling molecules, such as PI3K, NF-κB, activator protein 1 (AP-1), may contribute to the PPARγ-independent effects (Sawano et al., 2002; Takeda et al., 2001; Patel et al., 2005; Pereira et al., 2006; Bianchi et al., 2014). Studies also showed that 15d-PGJ_2_ induced a variety of cellular responses including activation of MAPKs (Kim J. et al., 2008; Lim et al., 2007), inactivation of p53 (Straus and Glass, 2007) and modulation of COX-2 (Kim E. et al., 2008) via PPARγ-independent mechanisms. The PPARγ-independent induction of COX-2 observed in our study likely stems from the unique chemical properties of 15d-PGJ_2_. Unlike synthetic thiazolidinediones, 15d-PGJ_2_ possesses a reactive α, β-unsaturated carbonyl moiety within its cyclopentenone ring. This structural feature renders it highly electrophilic, enabling the formation of covalent adducts with nucleophilic cysteine residues of cellular proteins via Michael addition (Tontonoz et al., 1994; Nguyen, 2006).

We postulate that at micromolar concentrations, 15d-PGJ_2_ imposes “electrophilic stress” on mesangial cells, potentially by depleting intracellular glutathione (GSH) or directly modifying redox-sensitive signaling molecules (such as Keap1 or IKK kinases). This is consistent with previous findings that 15d-PGJ_2_ upregulates COX-2 via a thiol-sensitive mechanism (Zhang et al., 2020) and involves reactive oxygen species (ROS) generation (Kitz et al., 2011). However, direct measurements of GSH levels and ROS production in our mesangial cell system were not performed in the current study, and thus this proposed mechanism remains to be experimentally validated in our experimental context. It is also worth noting that the 10 μM concentration of 15d-PGJ_2_ used in this study, while higher than physiological circulating levels, is consistent with concentrations commonly employed in mechanistic studies of 15d-PGJ_2_ in mesangial cells. Sawano et al. (2002) demonstrated PPARγ-independent inhibition of IL-1β-induced COX-2 expression using 10 μM 15d-PGJ_2_ in rat mesangial cells (Sawano et al., 2002), and Reyes-Martin et al. (2008) showed that 10 μM 15d-PGJ_2_ upregulates COX-2 expression in human mesangial cells through a thiol-sensitive mechanism (Reyes-Martin et al., 2008). Thus, the concentration used in our study is appropriate for investigating the mechanistic question of PPARγ dependence, which was the primary objective of this work. However, we acknowledge that whether lower, nanomolar concentrations (e.g., 0.1–1 μM) induce COX-2 remains an open question for future study.

This chemical reactivity operates distinctly from the classical ligand-receptor interaction, thereby explaining why genetic ablation or pharmacological blockade of PPARγ failed to abrogate COX-2 upregulation. The concept of “metabolic reprogramming”—whereby a pathological stimulus fundamentally reshapes cellular metabolic pathways—has emerged as a key framework for understanding how lipid mediators influence cellular function (Wang et al., 2026). Given this unique mode of action, this electrophilic stress may converge on multiple intracellular signaling hubs to drive COX-2 transcription. For instance, redox imbalance could modulate the lipid phosphatase PTEN, thereby relieving its brake on the PI3K/Akt axis, a pathway known to promote COX-2 expression (Takeda et al., 2001; Kim E. et al., 2008; An et al., 2022). Downstream of such kinase cascades, the activation of transcription factors like STAT6 (Jiao et al., 2021a; Jiao et al., 2021b) and SOX4 (Du et al., 2026), alongside epigenetic remodeling by the histone demethylase JMJD3 (An et al., 2023), may cooperatively facilitate rapid and robust COX-2 mRNA induction. Furthermore, as a danger signal, 15d-PGJ_2_ may engage innate immune sensors such as the cGAS-STING pathway (Jiao et al., 2025) or the NLRP3 inflammasome (Li et al., 2025), both potent inducers of inflammatory gene expression and PGE_2_ production. Given that 15d-PGJ_2_ extensively modifies cytoskeletal proteins (Stamatakis et al., 2006), a connection to the Hippo pathway effector TEAD1 (Tran et al., 2025), which regulates mitochondrial function and prevents necroptosis and inflammation, also warrants consideration. Besides electrophilic stress, other receptor-independent mechanisms reported in other cell types—such as activation of membrane prostaglandin DP2/CRTH2 receptors, inactivation of MAPK phosphatases, or direct covalent modification of NF-κB signaling components—might also participate in 15d-PGJ_2_ signaling and warrant future exploration in mesangial cells. We fully acknowledge that these proposed alternative pathways remain speculative in the current study. Direct pharmacological or genetic targeting will be required in future investigations to establish their precise roles. Thus, 15d-PGJ_2_ acts as a dual-signaling molecule: a receptor agonist at low concentrations and a chemical stressor inducing pro-inflammatory cascades at higher concentrations, embodying the “dual-pathway” model that distinguishes systemic receptor-mediated actions from local tissue-level effects (Zhai et al., 2026). It is important to note that the increased PGE_2_ production observed in our study is attributed primarily to the upregulation of COX-2 expression, as evidenced by the corresponding increases in COX-2 mRNA and protein levels. However, we cannot entirely exclude the possibility that 15d-PGJ_2_ may also modulate COX-2 enzymatic activity or the expression/activity of terminal PGE_2_ synthases, such as microsomal prostaglandin E synthase-1 (mPGES-1) and mPGES-2, which have been shown to be expressed in kidney and to play important roles in PGE_2_ production (Wang et al., 2018; Yang et al., 2006). While our data demonstrate that 15d-PGJ_2_-induced COX-2 expression occurs independently of PPARγ, whether 15d-PGJ_2_ affects these downstream enzymes or directly modulates COX-2 activity remains to be determined. Future studies are needed to dissect these potential contributions to the overall increase in PGE_2_ production. Nevertheless, our conclusion that 15d-PGJ_2_ induces COX-2 expression through a PPARγ-independent mechanism remains robust, as it is based directly on the measurement of COX-2 mRNA and protein levels.

From a translational perspective, these findings provide important insights for drug discovery (Fang et al., 2021): Future design of 15d-PGJ_2_ analogs should aim to uncouple PPARγ-dependent anti-inflammatory genomic actions from electrophilic COX-2 induction (Thakur et al., 2023); In diseases where PGE_2_ is pathogenic, high-dose 15d-PGJ_2_ therapy might inadvertently counteract its intended therapeutic benefits (Chen et al., 2023); Systematic *in vivo* dose-response studies are required to define a safe therapeutic window where PPARγ is activated without triggering COX-2-mediated pro-inflammatory cascades. Furthermore, given the complex cellular architecture of the glomerulus, 15d-PGJ_2_-induced COX-2/PGE_2_ in mesangial cells likely exerts paracrine effects on neighboring podocytes and endothelial cells. A limitation of the present study is the exclusive use of monocultured mesangial cells. Future investigations utilizing glomerulus-on-a-chip, cell co-culture systems, or single-cell transcriptomics will be essential to elucidate how mesangial-derived prostaglandins shape the integrated glomerular microenvironment.

In conclusion, the present study demonstrates that the endogenous prostaglandin metabolite 15d-PGJ_2_ markedly induces COX-2 expression and enhances PGE_2_ production in primary cultured mouse mesangial cells via a mechanism independent of PPARγ. Using multiple independent approaches, including pharmacological inhibition, dominant-negative interference, siRNA-mediated knockdown, and genetic deletion, we provide compelling evidence that this induction occurs independently of PPARγ. These findings highlight a receptor-independent mechanism through which electrophilic lipid mediators regulate inflammatory gene expression in renal cells. Given the critical role of COX-2-derived prostaglandins in renal inflammation and disease progression, our results suggest that the biological effects of 15d-PGJ_2_ in the kidney may involve both PPARγ-dependent and independent pathways. This dual mode of action should be considered when evaluating the therapeutic potential of prostaglandin metabolites and PPARγ-targeting strategies in renal diseases.

## Data Availability

The original contributions presented in the study are included in the article/supplementary material, further inquiries can be directed to the corresponding author.
